# Reduced serum neurotrophic factors and monoamine neurotransmitters in epilepsy patients with comorbid depression

**DOI:** 10.3389/fneur.2024.1480854

**Published:** 2024-10-15

**Authors:** Shulei Sun, Yuxiang Han, Xiaoyun Liu, Liling Yang, Tao Han, Youting Lin, Yabo Feng

**Affiliations:** Department of Neurology, Shandong Provincial Hospital Affiliated to Shandong First Medical University, Jinan, Shandong, China

**Keywords:** epilepsy, depression, brain-derived neurotrophic factor, glial cell line-derived neurotrophic factor, blood serum, cerebrospinal fluid, monoamine neurotransmitters

## Abstract

**Objective:**

This study aimed to investigate the roles of neurotrophic factors (NTFs), monoamine neurotransmitters, and inflammatory processes in the pathophysiology of the comorbidity of epilepsy and depression.

**Methods:**

A retrospective analysis was conducted with 57 epilepsy patients (PWE), 50 patients with epilepsy and comorbid depression (PWECD), and 47 healthy controls (HC) admitted between June 2020 and June 2024. Serum levels of brain-derived neurotrophic factor (BDNF), glial-derived neurotrophic factor (GDNF), 5-hydroxytryptamine (5-HT), norepinephrine (NE), dopamine (DA), interleukin-1β (IL-1β), and interleukin-6 (IL-6) were measured using enzyme-linked immunosorbent assay (ELISA). Additionally, BDNF and GDNF levels in cerebrospinal fluid (CSF) samples were analyzed from selected patients in the PWE and PWECD groups.

**Results:**

Serum BDNF levels were significantly lower in both PWE and PWECD groups compared to HC, while no differences between the former two groups. GDNF levels were lower in PWECD compared to HC, but not between PWE and HC. Serum 5-HT was significantly reduced in PWECD compared to both HC and PWE groups. No significant differences were observed in serum DA, NE, and IL-6 levels across the groups. Serum IL-1β levels were elevated in the PWECD group compared to the HC group. The Self-Rating Depression Scale (SDS) score negatively correlated with serum 5-HT and GDNF levels. In terms of predictive ability, serum BDNF demonstrated higher accuracy for the diagnosis of epilepsy [area under the curve, AUC = 0.701, 95% confidence intervals (95% CI) 0.601 ~ 0.801], while serum 5-HT was the best marker for predicting the development of depression in epilepsy patients (AUC = 0.727, 95% CI 0.632 ~ 0.821). No significant correlation was found between serum and CSF BDNF levels within the same subject (*r* = 0.155; *p* = 0.221; Spearman correlation), and CSF GDNF levels were too low to be clinically informative.

**Conclusion:**

The findings suggest the involvement of NTFs, monoamine neurotransmitters, and inflammatory processes in the pathogenesis of epilepsy and depression. Decreased serum BDNF levels correlate with epilepsy but not necessarily with comorbid depression, while serum GDNF and 5-HT show potential clinical value in diagnosing this comorbidity. However, the deficient levels of NTFs in CSF suggest a need for more sensitive detection methods.

## 1 Introduction

Epilepsy, a neurological disorder affecting over 70 million people worldwide, is frequently accompanied by cognitive and psychological challenges (1, 2). Depression is the most common psychiatric comorbidity in patients with epilepsy (PWE), affecting approximately one-third of individuals, especially those with temporal lobe epilepsy (TLE) and poorly controlled seizures (3, 4). Depression has been associated with the course of epilepsy (5), poor quality of life (than seizure frequency) (6), suicide attempt (7). An epidemiological study found that 9.3% of epilepsy patients had undiagnosed major depressive disorder (8). The inability to promptly identify and intervene for patients with epilepsy and comorbid depression (PWECD) greatly diminishes the quality of life for both patients with epilepsy and their caregivers (9, 10). The mechanisms causing PWECD are poorly understood, highlighting the urgent need to study further.

Neurotrophic factors (NTFs) are endogenous peptides or small proteins that regulate the proliferation, migration, differentiation, and survival of neural cells in the nervous system. They are essential for maintaining the function and structure of neurons, acting as critical molecular mediators in central synaptic plasticity (11). Dysregulation of NTFs, particularly brain-derived neurotrophic factor (BDNF), ciliary neurotrophic factor (CNTF), glial cell line-derived neurotrophic factor (GDNF), and nerve growth factor (NGF), has been implicated in various cerebral disorders, including epilepsy and depression. Therefore, NTFs can provide insights into the neuroplastic changes associated with these conditions.

Monoamine neurotransmitters, including dopamine (DA), 5-hydroxytryptamine (5-HT), and norepinephrine (NE), are fundamental to mood regulation. Dysfunction in these neurotransmitters is widely recognized as the biochemical basis for depression (11), and is associated with increased vulnerability to comorbid depression in individuals with epilepsy (12, 13). Additionally, neuroinflammation has emerged as a significant contributor to increased neural excitability and neurotransmitter imbalances (14), indicating a strong link between psychiatric symptoms and inflammation in epilepsy. The interaction between inflammation and neurotransmitters may be a common mechanism underlying both epilepsy and depression, warranting further research.

This study aimed to investigate the mechanisms underlying the co-occurrence of epilepsy and depression by comparing neurotrophic factors, monoamine neurotransmitters, and inflammatory markers among PWE, PWECD, and healthy controls (HC).

## 2 Materials and methods

### 2.1 Patients

**Figure 1:**
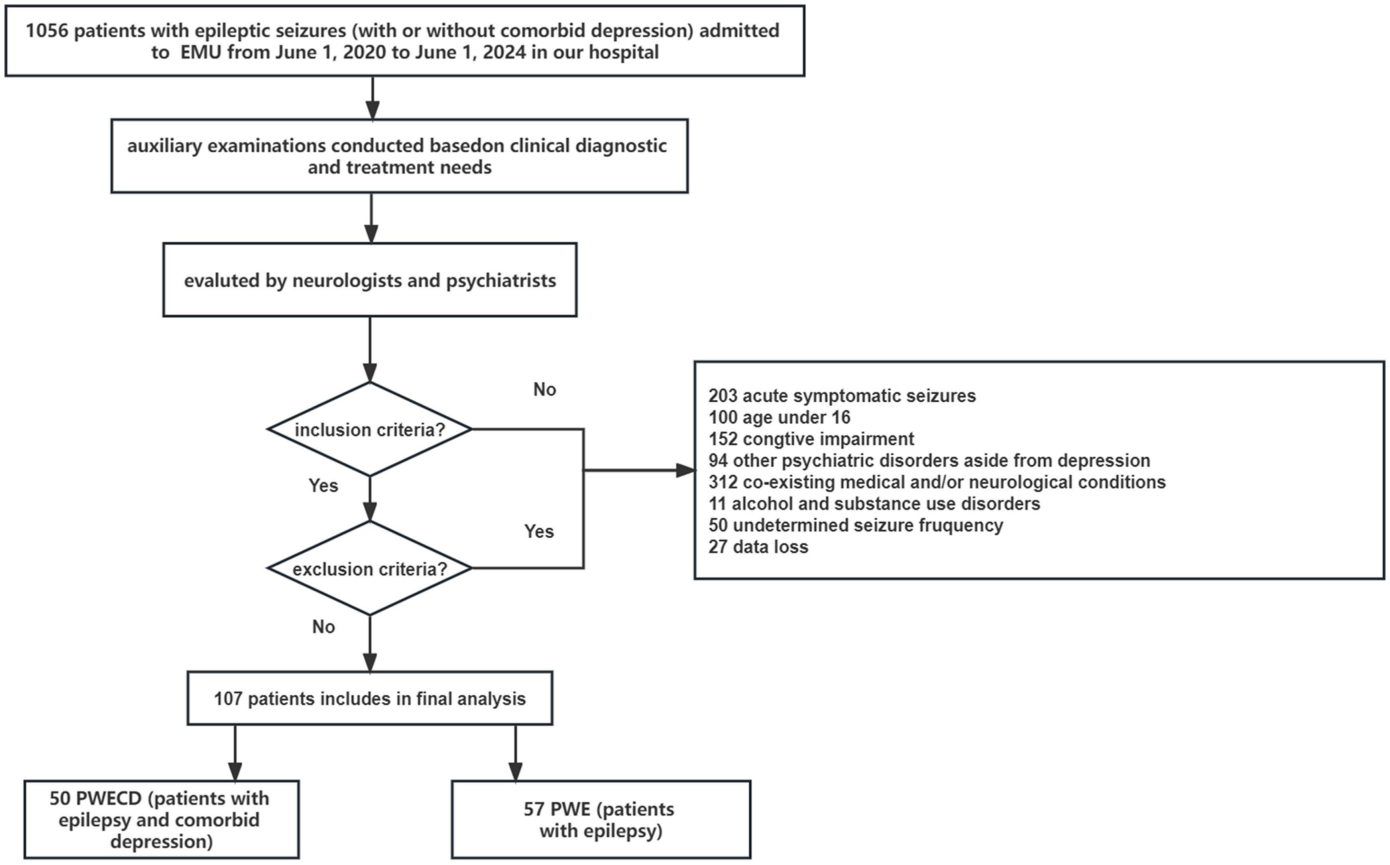
Flow diagram of the study.

We conducted a study which included patients with epileptic seizures who admitted to the epilepsy monitoring unit (EMU) of the Shandong Provincial Hospital affiliated with Shandong First Medical University from June 2020 to June 2024 (Figure 1). All the patients with epilepsy underwent electroencephalography (EEG) and magnetic resonance imaging (MRI) of the brain. Both neurologists and psychiatrists evaluated and diagnosed the patients to confirm the presence of epilepsy, with or without comorbid depression, based on the epilepsy criteria outlined by the International League Against Epilepsy (ILAE) (15–17), as well as the DSM-V (The Diagnostic and Statistical Manual of Mental Disorders, 5th edition) and the Mini-International Neuropsychiatric Interview (M.I.N.I.). The severity of depression was evaluated utilizing the Chinese iteration of the Zung scale (SDS, Self-Rating Depression Scale). Cognitive ability was evaluated using the Mini-Mental State Exam (MMSE). Normal MMSE scores were defined as >23 for junior high school and higher education literates, > 20 for primary school literates, and > 17 for illiterate subjects.

Our inclusion criteria were: (1) an established diagnosis of epilepsy; (2) aged 16 years or older; (3) proficiency in the Chinese language; and (4) the ability to provide informed consent and adhere to the study protocol. Participants were excluded if they had: (1) abnormal MMSE scores; (2) alcohol and substance use disorders, psychotic disorders, attention deficit hyperactivity, bipolar disorders (depression with mania or hypomania), manic/hypomanic episodes, or other psychiatric diseases; (3) co-existing medical and/or neurological conditions; and (4) undetermined seizure frequency.

Based on the presence of depression, participants were classified into two groups: PWE (patients with epilepsy) and PWECD (patients with epilepsy and comorbid depression). A total of 107 patients aged 16 years and above, including individuals with epilepsy (PWE, *n* = 57) and those with epilepsy comorbid with depression (PWECD, *n* = 50), were recruited. Additionally, 47 generally healthy volunteers of similar age and gender who exhibited no signs of mental disorders at the time of study enrollment were included as HC based on their medical records.

All patients did not receive treatment with antipsychotic drugs, antidepressants, or sedatives. Fifty-eight patients (54.2%) had been administered appropriate antiseizure medications (ASMs) prescribed by experienced epileptologists (standard treatment). The pharmacological treatment for epilepsy patients included first-generation antiepileptic drugs (such as carbamazepine and valproate sodium), second-generation antiepileptic drugs (like lamotrigine, levetiracetam, oxcarbazepine, zonisamide, and topiramate), and novel antiepileptic medications (such as lacosamide and perampanel).

### 2.2 Sample collection and measurement

- Cerebrospinal fluid (CSF) samples: Lumbar puncture was performed for clinical diagnostic purposes, and CSF samples were collected between 10–12 am to reduce potential circadian fluctuations in CSF composition. Routine laboratory analysis included evaluations for cell counts, total protein levels, and glucose concentrations. Extra 8–10 mL of CSF was promptly frozen at –80°C post-collection.
- Blood samples were obtained via venipuncture after an 8-h fast, left at around 21°C to 23°C for 1 h, then centrifuged (2000 g × 10 min) and divided into smaller portions. Serum samples were transferred to Eppendorf tubes and stored at –80°C.

Neurotrophic factor, neurotransmitter, and inflammatory factor concentrations were determined utilizing human enzyme-linked immunosorbent assays (ELISA) sourced from commercially available kits (ELISA BDNF, GDNF, IL-1β, IL-6 kits; Multi Sciences, Hangzhou, China; ELISA 5-HT, DA, NE kits; Elisa Lab, Wuhan, China). For BDNF and GDNF levels in CSF, 100 μL of the undiluted test sample was added to the sample well, deviating from the manufacturer’s instructions due to the low concentrations of neurotrophic factors in CSF.

### 2.3 Statistical analysis

Statistical analysis was conducted using SPSS Statistics 26.0 software and GraphPad Prism version 9.4.1 software (GraphPad Software, Inc., San Diego, CA, USA). Continuous variables were summarized using mean and standard deviation, while categorical variables were reported as median with interquartile range or percentage. Normality of distribution was assessed using the Shapiro–Wilk and Kolmogorov–Smirnov tests. Quantitative data were compared across multiple independent groups using either the ANOVA test or the Kruskal–Wallis test based on their distribution. *Post hoc* analysis involved the Tukey test or the Bonferroni test. Categorical variable comparisons were conducted using the Chi-square test or Fisher’s exact test. Spearman rank correlation test was performed to evaluate the correlation between the CSF and serum levels of BDNF. Multiple linear regression analysis was performed to identify the independent factors affecting SDS scores. The SDS score served as the dependent variable, while the independent variables included age, gender, and serum levels of BDNF, GDNF, 5-HT, and IL-1β. A significance level of *p* < 0.05 was considered statistically significant. Receiver operating characteristic (ROC) curve analysis was used to assess the diagnostic performance of the potential biomarkers for differentiating PWE from HC group, as well as PWECD from PWE group. For each biomarker, the area under the curve (AUC) was calculated to evaluate diagnostic accuracy. The optimal cutoff values for distinguishing between groups were determined based on the highest Youden index.

## 3 Results

### 3.1 Characteristics of the patients and the healthy control groups

The demographic and clinical information for PWE, PWECD, and HC groups are presented in Table 1. No significant differences were found in gender, age, BMI, educational background, employment status, smoking habits, and common laboratory parameters. None of the participants in the study were taking antidepressant medications. Twenty-nine patients (51%) in the PWE group and 20 patients (40%) in the PWECD group were not on ASMs.

**Table 1:** Patient and control group characteristics.

Parameter/Group	HC (*n* = 47)	PWE (*n* = 57)	PWECD (*n* = 50)	Statistics
Demographic data				
Age, years	35.51 ± 14.22	36.32 ± 12.96	34.60 ± 11.13	0.788
BMI, kg/m^2^	23.92 ± 3.85	23.95 ± 4.46	24.30 ± 4.12	0.883
Gender (male/female), %	49/51	58/42	46/54	0.436
Education (prim./sec./higher), %	9/51/40	7/51/42	6/60/34	0.490
Employment (−/+), %	38/62	49/51	44/56	0.542
Smoking (−/+), %	89/11	88/12	92/8	0.768
Clinical data				
Take antipsychotics or antidepressants, %	0	0	0	
Take ASMs (none/mono/polytherapy), %		51/37/12	40/38/22	0.166
Disease duration, months		3 (1.5,15)	3 (1,12)	0.873
SDS		33.75 (29,38)	63.75 (60,67.5)	<0.001
MMSE		28 (26,29)	27 (26,28)	0.937
Suicide ideation, %		7/93	26/74	0.009
Blood serum (plasma) indices				
BDNF, pg/mL	1439.62 ± 297.50	1222.34 ± 286.15	1127.70 ± 252.79	<0.001^a^
GDNF, pg/mL	1340.53 ± 317.53	1251.75 ± 248.25	1163.88 ± 236.84	0.006^b^
5-HT, ng/mL	115.99 ± 28.81	115.89 ± 23.69	94.88 ± 22.72	<0.001^c^
DA, pg/mL	82.95 ± 29.83	79.05 ± 26.06	73.05 ± 21.07	0.166
NE, pg/mL	148.63 ± 34.36	135.12 ± 29.55	138.39 ± 25.99	0.067
FT3, pmol/L	4.32 ± 0.60	4.24 ± 0.55	4.24 ± 0.54	0.724
FT4, pmol/L	12.05 ± 1.46	12.30 ± 1.41	12.27 ± 1.49	0.656
TSH, μIU/ml	1.46 ± 0.62	1.55 ± 0.63	1.55 ± 0.71	0.756
IL-1β	11.64 (10.85,12.72)	12.26 (10.93,13.31)	12.63 (11.19,14.49)	0.049^d^
IL-6	0.45 (0.38,0.65)	0.48 (0.38,0.71)	0.52 (0.36,0.68)	0.794
K, mmol/L	3.92 ± 0.25	3.93 ± 0.27	3.94 ± 0.27	0.887
Na, mmol/L	139.44 ± 1.60	139.66 ± 1.97	139.78 ± 2.40	0.708
Ca, mmol/L	2.38 ± 0.18	2.37 ± 0.14	2.38 ± 0.12	0.893
Total bilirubin, μmol/L	11.68 ± 5.03	11.29 ± 4.26	12.00 ± 4.49	0.727
Cholesterol, mmol/L	4.39 ± 0.73	4.19 ± 0.61	4.31 ± 0.94	0.411
Triglycerides, mmol/L	1.18 ± 0.63	1.18 ± 0.48	1.10 ± 0.36	0.656
Glucose, mmol/L	4.64 ± 0.53	4.59 ± 0.51	4.49 ± 0.43	0.333
Creatinine, μmol/L	56.73 ± 11.12	59.17 ± 11.51	57.3 ± 9.65	0.478
Seizure type				
Generalized onset motor, %		23	34	
Absence, %		5	6	
Focal onset aware seizure, %		19	10	0.514
Focal onset impaired awareness seizure, %		28	22	
Focal to bilateral tonic–clonic seizure, %		25	28	
Seizure frequency				
New-onset, %		14	6	
Absence in the last year, %		4	2	
Less than one per year, %		16	12	0.358
1–3 per month, %		47	46	
More than one per week, %		19	34	

Data are presented as the mean ± standard deviation (M ± SD) or the median with the interquartile range (M [Q1; Q3]); Differences between groups with quantitative data were assessed using one-way ANOVA or the Kruskal–Wallis test (with post hoc Tukey or Bonferroni test), and the Mann–Whitney U test was assessed for datasets composed of two groups. The chi-square test or Fisher’s exact test was used for qualitative data. Latter data that remained significant are depicted in bold (p values) in the Table with superscript letters, which are explained below. ^a^Significant differences: HC and PWE: < 0.001, HC and PWECD: *p* < 0.0001. ^b^Significant difference: HC and PWECD = 0.004. ^c^Significant differences: HC and PWECD < 0.001, PWE and PWECD < 0.0001. ^d^Significant differences: HC and PWECD = 0.043. ASMs, anti-seizure medications; SDS, self-rating depression scale; MMSE, mini-mental state examination; BDNF, brain-derived neurotrophic factor; GDNF, glial-derived neurotrophic factor; 5-HT, 5-hydroxytryptamine; DA, dopamine; NE, norepinephrine; FT3, free triiodothyronine; FT4, free thyroxine; TSH, thyroid-stimulating hormone; IL-1β, Interleukin-1β; IL-6, Interleukin-6; PWE, patients with epilepsy; HC, healthy controls; PWECD, patients with epilepsy and comorbid depression.

The PWECD group had higher levels of depressed mood and suicidal ideation compared to PWE, based on the SDS score. Cognitive levels (MMSE scale) showed no significant differences between PWE and PWECD. Seizure type did not impact cognitive and depression levels (Kruskal-Wallis test; for SDS, *p* = 0.403; for MMSE, *p* = 0.175), and seizure frequency did not impact cognitive and depression levels (Kruskal-Wallis test; for SDS, *p* = 0.630; for MMSE, *p* = 0.512).

### 3.2 Neurotrophic factors in serum and CSF

**Figure 2:**
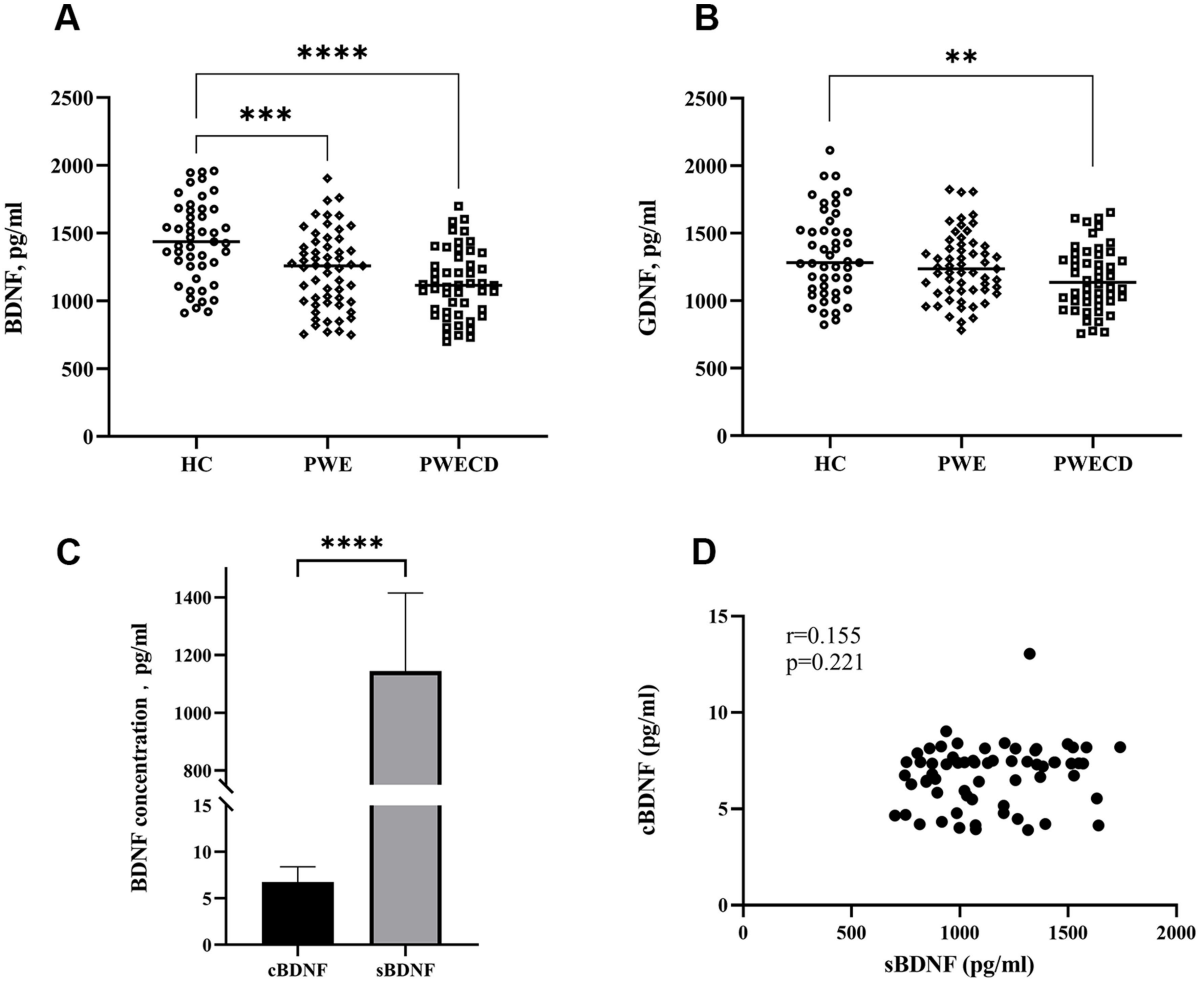
Neurotrophic factors in blood serum of PWE, PWCED, and the HC group: **(A)** BDNF; **(B)** GDNF. BDNF in biological fluids (cerebrospinal fluid and serum) of epilepsy patients with or without comorbid depression **(C,D)**. One-way ANOVA (with *post hoc* Tukey test) for Neurotrophic factors was used to compare multiple unrelated groups. Differences in BDNF between serum and CSF were assessed by the Wilcoxon test. **p < 0.01, ***p < 0.001, ****p < 0.0001.

Serum BDNF levels were significantly lower in the PWE and PWECD groups compared to HC, with no difference between the PWE and PWECD groups (Figure 2A; Table 1). Serum GDNF levels were significantly lower in PWECD than HC, with no difference between the PWE and HC groups and between the PWE and PWECD (Figure 2A; Table 1).

The CSF BDNF levels did not show significant differences between PWE and PWECD (Supplementary Table S1). There was an approximately 150-fold increase in the serum BDNF levels compared to the CSF levels (Figure 2C). No significant correlation was found between the serum and CSF BDNF levels within the same subject (*r* = 0.155; *p* = 0.221; Spearman correlation) (Figure 2D).

Although highly sensitive assays were employed (refer to the Sample collection and measurement section), CSF levels of GDNF were below the detection limit, providing no data to demonstrate its role in the central nervous system.

### 3.3 Monoamine neurotransmitters

**Figure 3:**
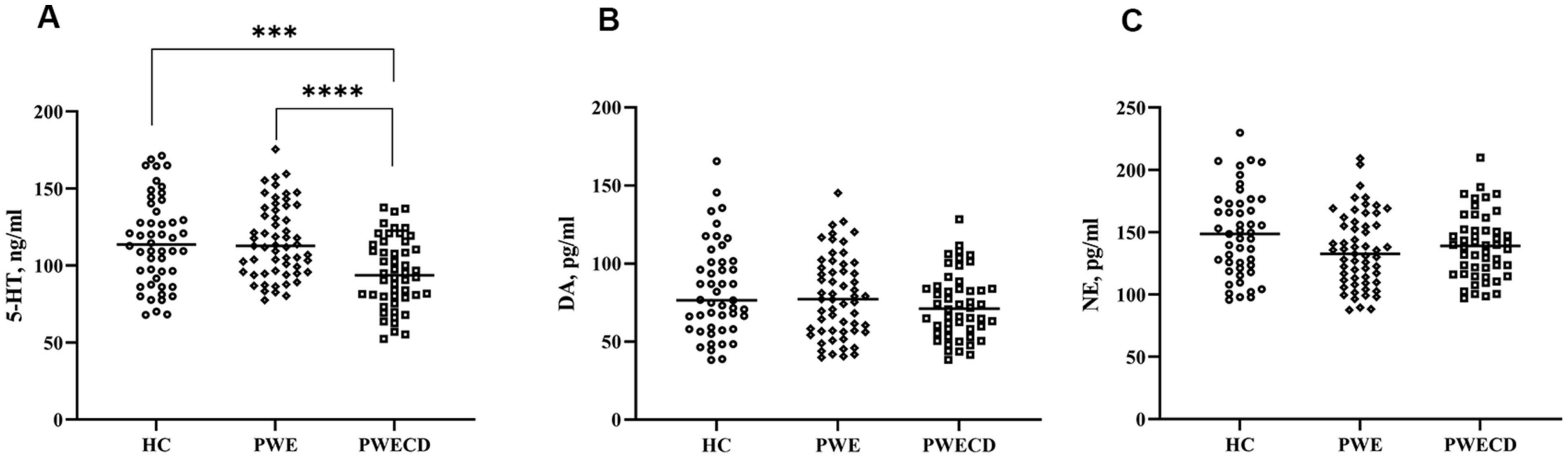
Monoamine neurotransmitters in blood serum of PWE, PWCED, and the HC group: **(A)** 5-HT; **(B)** DA; **(C)** NE. One-way ANOVA (with *post hoc* Tukey test) for Monoamine neurotransmitters was used to compare multiple unrelated groups. ***p < 0.001, ****p < 0.0001.

Serum levels of 5-HT were significantly lower in the PWECD group compared to both the HC and PWE groups, with no difference between HC and PWE groups (Figure 3A; Table 1). Serum DA and NE levels showed no significant differences among the three groups (Figures 3B,C; Table 1).

### 3.4 Inflammatory factors

**Figure 4:**
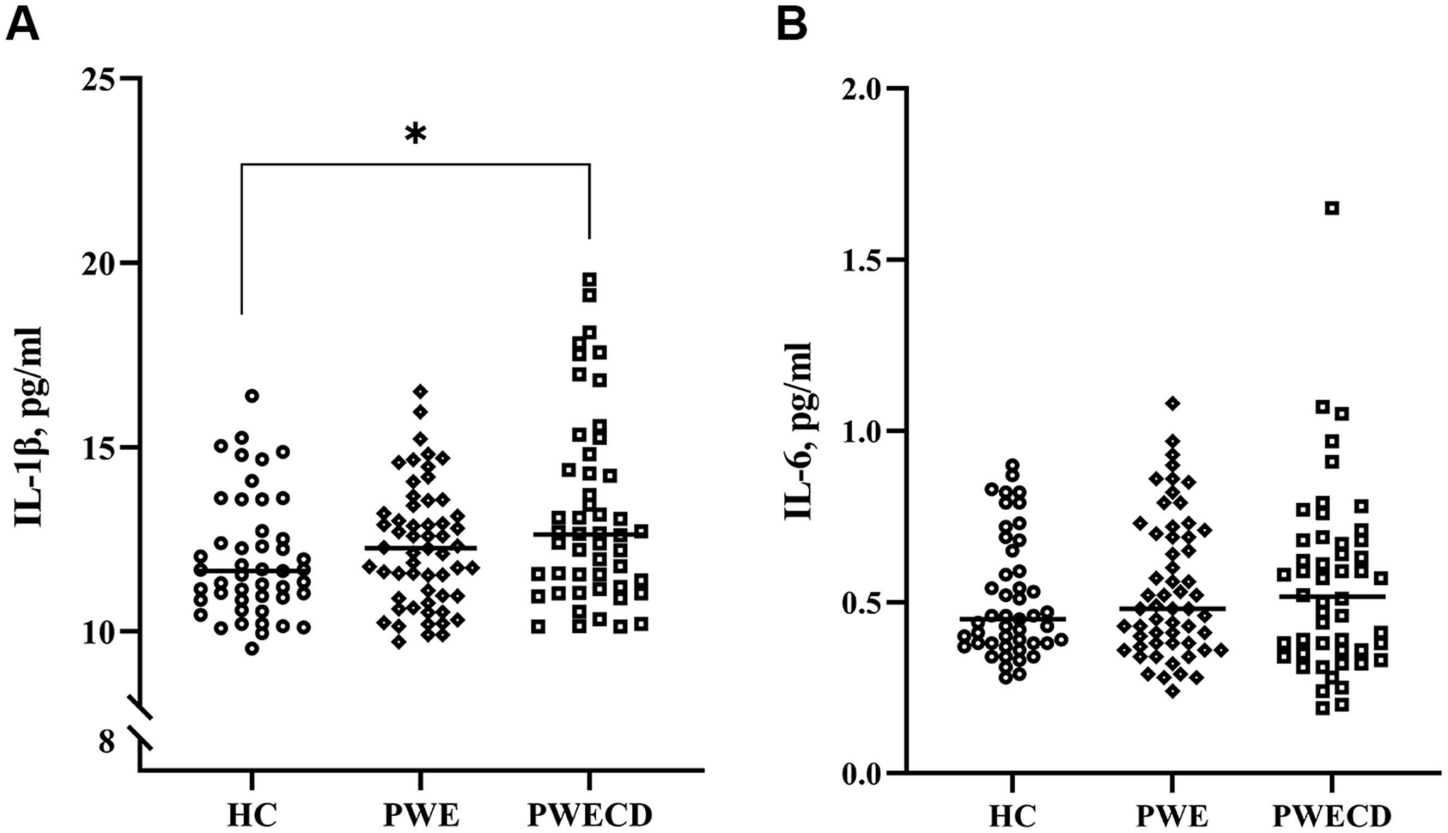
Inflammatory factors in blood serum of PWE, PWCED, and the HC group: **(A)** IL-1β; **(B)** IL-6. the Kruskal-Wallis test (with *post hoc* Bonferroni test) for IL-1β was used to compare multiple unrelated groups. *p < 0.05.

Serum levels of IL-1β were significantly elevated in the PWECD group compared to HC (Figure 4A; Table 1), while there was no significant difference between the PWE and HC groups or between the PWE and PWECD groups. No significant differences were observed in IL-6 levels among the three groups (Figure 4B; Table 1).

### 3.5 Multiple linear regression analysis for the SDS

Table 2 listed the details of Multiple Linear Regression Analysis. After adjusting for age and gender, multiple linear regression analysis demonstrated that both 5-HT and GDNF were independent factors affecting SDS score among all 107 epilepsy patients (Beta = −0.345, *p* < 0.001; Beta = −0.228, *p =* 0.042, respectively). Age, gender, BDNF and IL-1β had no significant impacts on the SDS score. Based on the standardized coefficient, there was a significant negative correlation between serum 5-HT and SDS scores, as well as between serum GDNF and SDS scores.

**Table 2:** Multiple linear regression analysis for the SDS.

Parameters	*B*	SEM	Standardized coefficient (Beta)	*p*-value	VIF
Age	−0.161	0.120	−0.117	0.182	1.053
Gender	2.628	2.882	0.079	0.364	1.046
BDNF	0.002	0.007	0.030	0.794	1.880
GDNF	−0.016	0.008	**−0.228**	**0.042**	1.719
5-HT	−0.227	0.062	**−0.345**	**<0.001**	1.248
IL-1β	1.204	0.716	0.154	0.096	1.172

R^2^ = 0.285. SEM, standard error of mean; VIF, variance inflation factor; BDNF, brain-derived neurotrophic factor; GDNF, glial-derived neurotrophic factor; 5-HT, 5-hydroxytryptamine; IL-1β, Interleukin-1β; SDS, Self-Rating Depression Scale. Statistically significant beta values are indicated in bold.

### 3.6 Optimal cutoffs of BDNF, GDNF, 5-HT, NE, DA, IL-1β, IL-6 predictors for the probability assessment of PWE, PWECD

**Figure 5:**
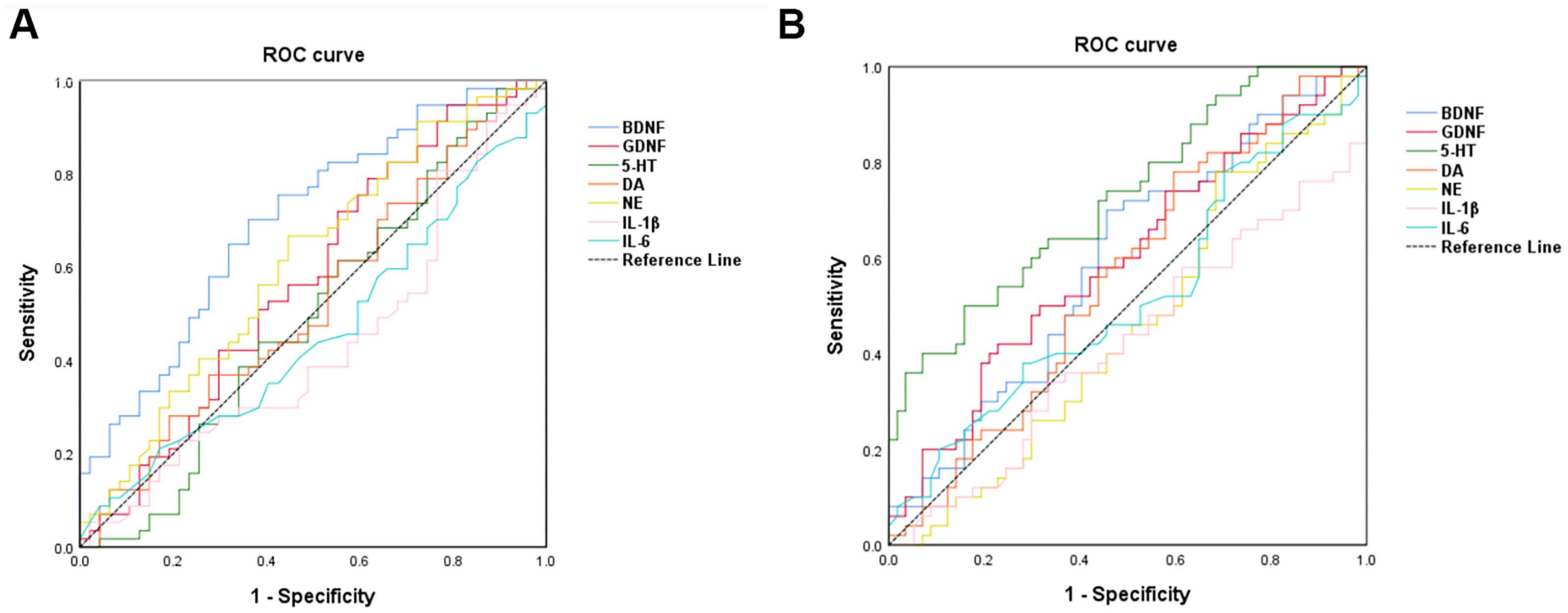
Receiver operating characteristic curve analysis of the 7 serum markers. **(A)** BDNF was the best marker to discriminate PWE from HC (area under the curve = 0.701). **(B)** 5-HT was the best marker to discriminate PWCED from PWE (area under the curve = 0.727).

The diagnostic performance of various biomarkers was evaluated using ROC curve analysis. Among these, BDNF demonstrated the highest efficacy in distinguishing PWE from HC, with an area under the curve (AUC) of 0.701 (*p* < 0.001) and an optimal cutoff value of <1360.16, as shown in Figure 5A and Table 3. Additionally, serum 5-HT levels were found to be the most effective in distinguishing PWECD from PWE, with an AUC of 0.727 (*p =* 0.001) and an optimal cutoff value of <93.09, as shown in Figure 5B and Table 4.

**Table 3:** Clinical utility indexes of neurotrophic factors, monoamine neurotransmitters, and inflammatory factors for predicting patients with epilepsy.

Serum indices	Optimal cut-off	Sensitivity (%)	Specificity (%)	Youden index
BDNF	1360.16	70.20	63.80	0.340
GDNF	1428.23	78.90	38.30	0.172
5-HT	162.00	98.20	10.60	0.089
DA	65.36	36.80	72.30	0.092
NE	144.21	66.70	55.30	0.220
IL-1β	13.57	80.70	23.40	0.041
IL-6	0.3	8.80	95.70	0.045

BDNF, brain-derived neurotrophic factor; GDNF, glial-derived neurotrophic factor; 5-HT, 5-hydroxytryptamine; DA, dopamine; NE, norepinephrine; IL-1β, Interleukin-1β; IL-6, Interleukin-6.

**Table 4:** Clinical utility indexes of neurotrophic factors, monoamine neurotransmitters, and inflammatory factors for predicting epilepsy comorbid with depression.

Serum indices	Optimal cut-off	Sensitivity (%)	Specificity (%)	Youden index
BDNF	1236.64	70.00	54.40	0.244
GDNF	1064.78	42.00	77.20	0.192
5-HT	93.09	50.00	84.20	0.342
DA	85.58	78.00	40.40	0.184
NE	152.35	78.00	31.60	0.096
IL-1β	11.56	34.00	66.70	0.007
IL-6	0.395	38.00	71.90	0.099

BDNF, brain-derived neurotrophic factor; GDNF, glial-derived neurotrophic factor; 5-HT, 5-hydroxytryptamine; DA, dopamine; NE, norepinephrine; IL-1β, Interleukin-1β; IL-6, Interleukin-6.

## 4 Discussion

In this study, we revealed the significant differences in serum BDNF, GDNF, 5-HT, and IL-1β among the HC, PWE, and PWECD groups, along with the negative correlation with depression severity in serum GDNF and 5-HT levels, supporting their role in epilepsy and comorbid depression.

It is not surprising to find decreased serum levels of GDNF and 5-HT in the PWECD group compared to the control. GDNF functions by binding to its receptor GFR-α1 and activating the c-Ret tyrosine kinase signaling pathway, which supports the development, survival, and differentiation of dopaminergic, serotonergic, and GABAergic neurons (18–20). Given our results showing lower serum GDNF levels in the PWECD group, we hypothesize that GDNF deficiency-induced neurotransmitter disruption may be a key mechanism in the pathogenesis of depression comorbid with epilepsy. Recent studies have found that reduced levels of GDNF in plasma (21), serum (22), and lacrimal fluid (23) are significant for diagnosing and predicting depressive disorders, providing further support for our hypothesis. Similarly, 5-HT is widely recognized as a crucial neurotransmitter for mood regulation and is a classical target of antidepressant therapies, helping explain our findings of significantly decreased 5-HT levels in the PWECD group. Furthermore, the ROC curve analysis demonstrated the ability to distinguish between PWE and PWECD groups, highlighting the potential of 5-HT as a biomarker for identifying depression comorbid with epilepsy.

Interestingly, while BDNF levels also decreased in both PWE and PWECD groups compared with the control, there was no difference between the PWE and PWECD groups. This observation is consistent with previous studies (24–26), which have also reported decreased BDNF levels in the serum of epilepsy patients, aligning with our results and reinforcing the association between BDNF reduction and seizure activity. Although reduced serum BDNF levels have been linked to be associated with an increased risk of developing major depression and suicidal ideation (27, 28), findings are inconsistent across all studies (29, 30), and the underlying mechanisms remain unclear. It is possible that central BDNF levels, rather than serum BDNF, may be more closely correlated with mood disorders and suicidality, as studies have demonstrated lower BDNF levels in the prefrontal cortex and hippocampus of individuals with major depression and those who have died by suicide (31–33). Despite our attempt to assess BDNF changes in CSF, we found no significant differences between the PWE and PWECD groups and could not establish a correlation between serum and CSF BDNF levels. Based on our current findings and literature review (34), it remains unclear whether the reduction in serum BDNF levels associated with recurrent seizures could be a mechanism contributing to the development of PWECD. This question warrants further investigation.

Preclinical studies have shown that seizures can be suppressed by the overexpression of BDNF or GDNF in the hippocampus or cerebral cortex in animal models of epilepsy, suggesting that the lack of these NTFs may play a role in epileptogenesis (35–37). However, due to the presence of the blood–brain barrier (BBB), serum NTF levels do not necessarily reflect NTFs levels in brain tissue. NTFs are released from both peripheral tissues and the central nervous system. For instance, in the CNS, neurons, astrocytes, and microglia are the main producers of BDNF, whereas BDNF is also extensively synthesized by peripheral cells, such as megakaryocytes in endothelial cells (38), bone marrow (39), and various immune cells (40), including B and T lymphocytes and monocytes. The relationship between NTFs levels in the CNS and peripheral systems, as well as their clinical relevance to CNS disorders, remains unclear. Early studies in rodents suggested the use of peripheral measurements as proxies for CNS levels (41, 42). However, recent research has challenged this assumption (43–45). Our study contributes to this discussion by demonstrating the lack of a clear correlation between CSF and serum NTFs. Using ELISA to measure BDNF and GDNF in undiluted CSF, we found that GDNF levels in CSF were too low to detect, and BDNF levels, though measurable, were more than 100 times lower than in serum. These findings highlight the challenges of relying on CSF-based BDNF measurements in clinical practice (46). This significant discrepancy may be attributed to the dual synthesis of NTFs in both the central and peripheral systems, as well as the effects of the BBB (47). Therefore, further investigation is need to clarify the implications of decreased peripheral NTF levels for diagnosing and managing nervous system disorders.

Although our results underscore the significant roles of NTFs in epilepsy or depression, their involvement in other neuropsychiatric disorders, particularly neurodegenerative diseases such as Parkinson’s disease (PD), Alzheimer’s disease (AD), Huntington’s disease (HD), and amyotrophic lateral sclerosis (ALS), is also receiving growing attention (48). GDNF has been extensively studied in PD, where reduced serum levels are associated with the degeneration of dopaminergic neurons. GDNF supports the survival and differentiation of these neurons, and clinical trials involving direct brain delivery of GDNF have shown promise in halting or even reversing motor symptoms in PD patients (49, 50). Similarly, BDNF is vital for synaptic plasticity and memory formation, playing a key role in diseases like HD and AD. BDNF supports various neuron types, including cholinergic and dopaminergic neurons, and its depletion has been linked to cognitive decline and motor dysfunction (51, 52). Both GDNF and BDNF are increasingly implicated in a broader range of neurodegenerative and neuropsychiatric disorders, and understanding their interactions with disease pathology could position them as vital therapeutic targets.

Neuroinflammatory changes have been identified in cerebrospinal fluid (CSF) and brain samples from patients with epilepsy, especially those with refractory epilepsy (53, 54). The pathogenesis may involve prolonged activation that leads to local microgliosis and astrogliosis, as well as disruption of the blood–brain barrier (BBB), contributing to chronic neuroinflammation and increased synaptic excitability (55–57). Additionally, sterile neuroinflammation is suggested as a fundamental mechanism underlying various psychiatric disorders and PWECD as well (56). We found that IL-1β levels were significantly elevated in the PWECD group, further supporting the hypothesis of neuroinflammation’s role. The reason for highlighting neuroinflammation is that if we aim to investigate the distribution of NTFs in serum and CSF further and confirm their roles in PWECD and PWE, it is essential to take into consideration the impact of chronic inflammation and the potential effects of BBB disruption. Understanding these factors will be crucial for elucidating the mechanisms of NTFs in these conditions. However, the current results do not yet demonstrate a direct relationship between these factors.

This study has the following limitations. First, it predominantly provides insights into a clinical phenomenon through observational outcomes and further studies are needed to investigate the underlying mechanisms in detail. (1) Employing more sensitive detection methods to accurately measure low levels of NTFs in cerebrospinal fluid (CSF); (2) Paying attention to potential neuroinflammatory mechanisms and assessing whether changes in blood–brain barrier permeability may affect the distribution of NTFs in the CSF and serum; and (3) Conducting longitudinal studies and intervention trials to clarify the temporal and causal relationships between these biomarkers and comorbid depression in epilepsy patients, and to seek more reliable evidence validating the clinical significance of these biomarkers in disease pathogenesis and prognosis.

## 5 Conclusion

In conclusion, this study identified significant differences in serum BDNF, GDNF, 5-HT, and IL-1β among the HC, PWE, and PWECD groups, suggesting their involvement in epilepsy and comorbid depression. The negative correlation between GDNF, 5-HT and the SDS score highlights their potential as biomarkers for PWECD. These findings enhance our understanding of PWECD and may pave the way for new diagnostic and therapeutic approaches in the future.

## Data Availability

The raw data supporting the conclusions of this article will be made available by the authors, without undue reservation.
