# Supplementary material for: Reduced serum neurotrophic factors and monoamine neurotransmitters in epilepsy patients with comorbid depression
Source: Front Neurol. 2024 Oct 15;15:1480854. doi: 10.3389/fneur.2024.1480854 (PMC11518734; doi:10.3389/fneur.2024.1480854)
Supplement: Supplementary file 1 [file Table_1.DOCX]

| **Parameter/** | **PWE** | **PWCED** | **Statistics** |
| --- | --- | --- | --- |
| **Group** | **(n=34)** | **(n=30)** |  |
| Age, years | 36.82 ± 12.73 | 34.30 ± 12.51 | 0.428 |
| gender (male/female), % | 59/41 | 47/53 | 0.331^a^ |
| BMI, kg/m^2^ | 23.74 ± 4.58 | 24.50 ± 4.25 | 0.494 |
| **Serum indices** |  |  |  |
| GDNF, pg/ml | 1213.30 ± 245.63 | 1176.72 ± 250.37 | 0.558 |
| BDNF, pg/ml | 1172.16 ± 291.52 | 1112.55 ± 245.69 | 0.383 |
| **CSF indices** |  |  |  |
| BDNF, pg/ml | 7.33 (5.36, 7.49) | 7.34 (5.75, 8.13) | 0.527 |
| total cell count, 10^6^/L | 1.5 (1.0, 2.0) | 2.0 (1.0, 3.0) | 0.498 |
| chloride, mmol/L | 123.40 ± 1.89 | 122.99 ± 2.52 | 0.458 |
| glucose, mmol/L | 3.66 (3.47, 4.07) | 3.61 (3.34, 3.99) | 0.532 |
| protein, g/L | 0.35 (0.29, 0.49) | 0.38 (0.34, 0.53) | 0.281 |

**Supplementary Table 1.** CSF and serum neurotrophic factor levels

a. Fisher’s exact test.
